# Exploring the microbial landscape: uncovering the pathogens associated with community-acquired pneumonia in hospitalized patients

**DOI:** 10.3389/fpubh.2023.1258981

**Published:** 2023-12-13

**Authors:** Karin Hansen, Linda Yamba Yamba, Lisa Wasserstrom, Elisabeth Rünow, Tommy Göransson, Anna Nilsson, Jonas Ahl, Kristian Riesbeck

**Affiliations:** ^1^Clinical Microbiology, Department of Translational Medicine, Faculty of Medicine Lund University, Malmö, Sweden; ^2^Infectious Diseases, Department of Translational Medicine, Faculty of Medicine Lund University, Malmö, Sweden; ^3^Clinical Microbiology, Infection Control and Prevention, Laboratory Medicine, Lund, Sweden

**Keywords:** CAP, community acquired pneumonia, ECAPS, *Haemophilus influenzae*, influenza virus, lower respiratory tract infection, *Mycoplasma pneumoniae*, *Moraxella catarrhalis*

## Abstract

**Objectives:**

This study aimed to investigate the etiology, clinical features, and outcomes of community-acquired pneumonia (CAP) in adults. Understanding the causative pathogens is essential for effective treatment and prevention.

**Design:**

Between 2016–2018, 518 hospitalized adults with CAP and 241 controls without symptoms were prospectively enrolled. Urine samples were collected for pneumococcal urinary antigen tests and nasopharyngeal swabs for viral and bacterial analysis, combined with routine diagnostic care.

**Results:**

Among the included CAP patients, *Streptococcus pneumoniae* was the most common pathogen, detected in 28% of patients, followed by *Haemophilus influenzae* in 16%. Viruses were identified in 28%, and concurrent viruses and bacteria were detected in 15%. There was no difference in mortality, length of stay, or symptoms at hospitalization when comparing patients with bacterial, viral, or mixed etiologies. Among the control subjects without respiratory symptoms, *S. pneumoniae*, *H. influenzae*, or *Moraxella catarrhalis* were detected in 5–7%, and viruses in 7%.

**Conclusion:**

*Streptococcus pneumoniae* emerged as the predominant cause of CAP, followed closely by viruses and *H. influenzae*. Intriguingly, symptoms and outcome were similar regardless of etiology. These findings highlight the complexity of this respiratory infection and emphasize the importance of comprehensive diagnostic and treatment strategies.

**Clinical Trial Registration**: ClinicalTrials.gov, identifier [NCT03606135].

## Introduction

Community-acquired pneumonia (CAP) remains a leading cause of infectious disease-related death (1), despite preventative measures aimed at combating CAP. These measures include pneumococcal vaccination of at-risk groups, yearly vaccinations for seasonal influenza viruses, and, since 2021, SARS-CoV-2 vaccination. In Sweden, the *Haemophilus influenzae* type b (Hib) vaccine has been included in the child immunization program since the early 1990s and conjugated pneumococcal vaccines (PCV) were introduced in 2009. Interestingly, PCV has led to a reduction of all-cause CAP in children, but a similar decrease has not been shown in older adults (2).

*Streptococcus pneumoniae* has been regarded as the primary etiology of CAP but other pathogens, such as *H. influenzae* and atypical bacteria, are also important. In addition, viruses have been recognized as the sole cause of CAP, and combined etiologies have been observed to a higher extent in patients with CAP treated in intensive care units (ICUs) (3, 4).

Understanding the etiology of CAP is crucial for accurate empirical antibiotic treatment, updating treatment guidelines, and future vaccine cost–benefit analyses. Currently, microbiological testing for CAP patients does not identify the etiology of most cases. However, incorporating molecular diagnostic methods can increase the detection rate of both viral and bacterial pathogens (5, 6). This is particularly important in an era of increasing antimicrobial resistance, as it offers the potential to de-escalate antibiotic treatment (6, 7). The aim of this study was to investigate the current etiology of CAP after the introduction of PCV in the child immunization program and to describe the clinical features based on detection of bacterial pathogens, viruses, or both.

## Methods

### Study design, setting, and patients

From September 2016 to September 2018, the prospective case–control study entitled “Etiology of community-acquired pneumonia in Sweden” (ECAPS) was conducted (ClinicalTrials.gov identifier: NCT03606135). The study was planned to cover two influenza seasons. This current study serves as a sub-analysis of the ECAPS cohort previously described by Hansen et al. (8). Patients aged 18 years or older presenting to the emergency department (ED) at Skåne University Hospital were screened for eligibility and included within 48 h of admission to the ED. The inclusion criteria consisted of having at least two out of ten predefined symptoms of respiratory tract infection, a radiological finding consistent with pneumonia according to the clinical radiologist on duty, and the ability to provide a urine sample. Exclusion criteria were hospitalization or pneumococcal vaccination within the last 30 days and previous enrolment in the study. A control group was continuously recruited throughout the study period at the Department of Orthopedics, within 48 h of admission. Exclusion criteria for the control group were symptoms of a respiratory tract infection within the past 14 days, or pneumococcal vaccination within 30 days prior to hospitalization.

### Microbiological testing

Samples from study patients were collected on clinical indication in routine care and analyzed using standard methods at the local Clinical microbiology laboratory (Laboratory Medicine Skåne). Samples from blood, nasopharynx, pleural fluid, and the lower respiratory tract were included. Urinary samples were analyzed for *Legionella pneumophila* antigen. Standard-of-care methods are further described in the Supplementary material. As per protocol, a nasopharyngeal (NP) flocked swab and a urine sample were collected within 48 h of admission. Multiplex real-time PCR was conducted on the NP samples to detect 14 viral agents (influenza A H1N1, influenza A H3N2, influenza B, enterovirus, rhinovirus, parechovirus, adenovirus, human metapneumovirus [hMPV], and coronaviruses [OC43, NL63, 229E]), as well as parainfluenza virus 1 to 3 and respiratory syncytial virus (RSV) A/B. In addition, real-time PCR was used to identify 6 bacterial pathogens (*S. pneumoniae, H. influenzae*, *Bordetella pertussis*/*parapertussis*, *Chlamydia pneumoniae*, and *Mycoplasma pneumoniae*). The PCR protocols and references can be found in the Supplementary material.

Urine specimens were tested at Pfizer’s Vaccines Research and Development Laboratory (Pearl River, NY). The testing involved a pneumococcal urinary antigen, BinaxNOW *S. pneumoniae*® (Abbott Diagnostics, Scarborough, ME), as well as two serotype-specific urinary antigen tests, [urine antigen detection; UAD 1 and 2, covering a total of 24 pneumococcal serotypes as described by Hansen et al. (8) (Supplementary material)]. Complete sampling was defined as including a blood culture, nasopharyngeal culture, UAD, BinaxNOW *S. pneumoniae*® test, and viral/bacterial real-time PCR analysis of 20 pathogens, as per protocol.

Two NP swabs and a urine sample were also collected from the control patient group. One NP swab was cultured for bacteria and the other swab analyzed with real-time PCR for the same 22 respiratory pathogens as the study patients. For PCR analysis every other sample was included to account for seasonal variance (*n* = 241). The urine samples were analyzed with the same urinary antigens as the study patients, BinaxNOW *S. pneumoniae*® and UAD 1 and 2.

### Data collection

Symptoms and previous vaccinations were registered by the study nurse through patient interviews. Auscultatory findings and medical history were collected from the patient’s chart.

### Disease severity and risk classification

The pneumonia severity index (PSI) was calculated upon study inclusion and used as a marker of severity. This continuous score is divided into 5 sub-classes based on 20 predictors (9), with class IV-V indicating a moderate-to-high risk of mortality. Case fatality rates (CFR) at 30 or 90 days were used to measure mortality.

### Statistical analyses

Statistical analysis was performed using RStudio 4.3.0 and SPSS version 29. For the comparison of proportions, we used either Pearson’s chi-squared test or Fisher’s exact test. Missing data were excluded from the analysis. In group comparisons of continuous parametric data, we employed ANOVA and *t*-tests, while the Kruskal-Wallis one way analysis of variance and Mann–Whitney U test were used for non-parametric data. A value of *p* below 0.05 was regarded as statistically significant, and we applied Bonferroni corrections where appropriate.

## Results

### Patient characteristics

We included 518 patients in the final analysis (Supplementary Figure S1), with a median age of 73 years (Table 1). Among them, 236 (46%) were females, and a minority of 13 (3%) resided in a nursing home. Fourteen patients (3%) were admitted to the ICU, and the case fatality rates (CFR) at 30 and 90 days were 4% (*n* = 21) and 8% (*n* = 43), respectively. Our control group (*n* = 241), matched for season, had a median age of 64 years, with 124 (52%) being females. Table 1 provides a detailed description of the CAP patient cohort and controls is described by Hansen et al. (8).

**Table 1:** Demography for CAP-patients and controls.

	CAP-patients	Controls	Missing data
*n* (%) or median [IQR]	518	241	Patients/ controls (*n*)
Age	73 [60–82]	64 [47–76]	0/0
Female sex	236 (45.6)	124 (51.5)	0/0
Nursing home resident	13 (2.5)	1 (0.4)	8/0
Smoker- current	97 (18.7)	36 (14.9)	1/1
Smoker - previous	234 (45.2)	66 (27.4)	1/1
BMI^a^	25 [22–29]	25 [23–28]	0/2
COPD	143 (28)	11 (4.6)	4/0
Asthma	47 (9.1)	10 (4.1)	0/0
Congestive heart disease	95 (18.3)	15 (6.2)	0/0
Coronary artery disease	135 (26.1)	19 (7.9)	0/0
Autoimmune disease	32 (6.2)	6 (2.5)	1/0
Diabetes	87 (16.8)	24 (10.0)	0/0
Liver disease	10 (1.9)	1 (0.4)	0/0
Immunosuppressive therapy^b^	65 (12.5)	9 (3.7)	2/0
Chronic kidney disease	47 (9.1)	4 (1.7)	1/0
Immunodeficiency^c^	3 (0.6)	2 (0.8)	1/0
Organ transplantation	5 (1.0)	2 (0.8)	1/0
Cancer - hematologic	19 (3.7)	2 (0.8)	2/0
Cancer solid tumor	106 (20.5)	30 (12.4)	1/0
Pneumococcal vaccine	56 (11)	.	29/0
Influenza vaccine	172 (35)	.	24/0
Antibiotic use 14 days prior to admission	105 (20.3)	.	2/0
PSI grade IV-V	262 (50.5)	.	0/0
CRB-65 3–4	14 (2.7)	.	0/0
Length of stay	5.0 [3–9]	.	0/0
ICU admission	14 (2.7)	.	0/0
Case fatality rate (CFR)			
30 days	21 (4.1)	.	0/0
90 days	43 (8.3)	.	0/0

a BMI – Body mass index; COPD - Chronic obstructive pulmonary disease; ICU – Intensive care unit; PSI – Pneumonia severity index.

b Prednisolone dose ≥ 10 mg/day (or equivalent), biological/immunomodulatory or chemotherapy drugs.

c Including primary immunodeficiency disorders, HIV and AIDS.

### Overall microbial detection

**Figure 1:**
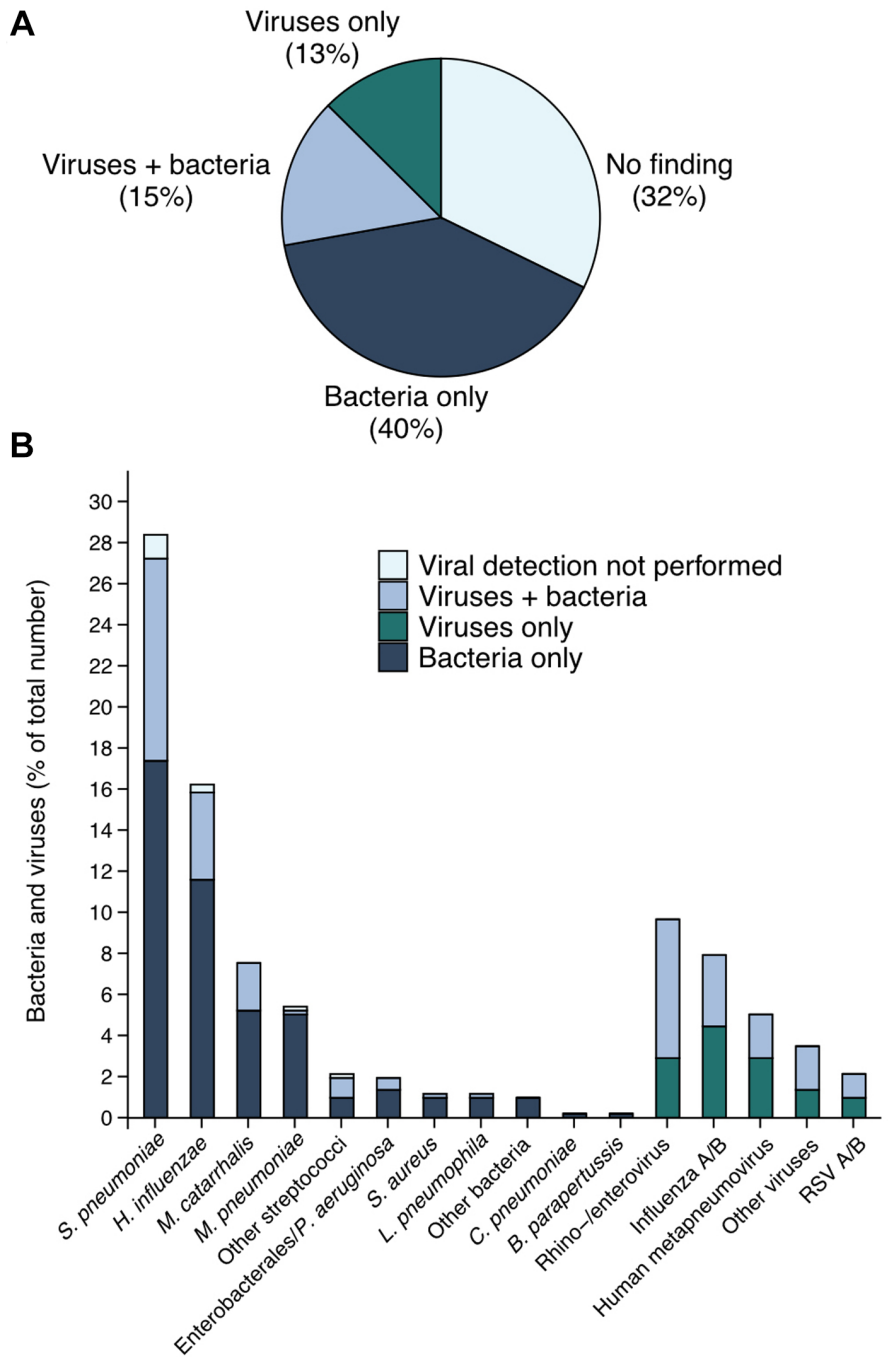
Etiology of CAP within the cohort. In **(A)** the pie chart shows the proportions of microbiological findings in the whole cohort. A minor part (23 individuals only) was not tested for viral pathogens; 2.1% in the group with bacteria only, and 2.3% in the group “no finding”. In **(B)** the bars in the figure represent bacteria or viruses indicated on the x-axis. Some patients had multiple pathogens detected. The group “Other bacteria” (*n* = 5) includes *Neisseria meningtidis, Fusobacterium nucleatum* and *Enterococcus faecalis*, whereas group labeled as “Other viruses” (*n* = 18) consists of parainfluenza virus 1 to 3, adenovirus and coronavirus (OC43, NL63, 229E). The group “Enterobacterales / *P. aeruginosa*” (*n* = 10) includes *Escherichia coli, Proteus Mirabilis, Enterobacter cloacae, Klebsiella pneumoniae* and *Pseudomonas aeruginosa*. The group “Other streptococci” (*n* = 11) includes β-hemolytic streptococci and *Streptococcus anginosus*.

One or more potential pathogens were detected by any method in 351 patients (68%). Among these, bacteria were found in 286 (55%), viruses in 144 (28%), and both bacteria and viruses in 79 (15%) patients. The proportions of microbiological findings are illustrated in Figure 1. The most common pathogens were *S. pneumoniae* (*n* = 147, 28%) followed by *H. influenzae* (*n* = 84, 16%), rhinovirus (9%), and influenza A/B (8%) (Supplementary Table S1). We detected multiple viruses in only two patients, but 79 (55%) of all viral cases also had a bacterial finding, with *S. pneumoniae* and rhinovirus being the most frequent combination. Thirty-eight patients (7%) had two or more identified bacteria, most commonly *S. pneumoniae* and *H. influenzae. Moraxella catarrhalis* was detected in 39 (8%) of CAP patients, of which 11 (28%) were in combination with another bacterium.

If we only consider cases where complete sampling was performed (*n* = 370, 71%) *S. pneumoniae* was detected in 30% (*n* = 113), *H. influenzae* in 17% (*n* = 63), *M. catarrhalis* in 9% (*n* = 35), and *M. pneumoniae* in 6% (*n* = 21). Co-detections are listed in Supplementary Table S2, and the detection in relation to the diagnostic method is shown in Supplementary Tables S3, S4.

The incidence of *S. pneumoniae* peaked during the winter seasons, as did *M. pneumoniae.* However, *H. influenzae* was not detected with the same seasonal variance. Influenza A dominated the winter season 2016–2017, and influenza B was dominant in 2017–2018. Rhino/enteroviruses were the only viruses detected during the summer periods (Supplementary Figure S2).

In contrast to patients with CAP, a bacterium was detected in 20% (47/241) of our controls with an equal distribution of *S. pneumoniae, H. influenzae* and *M. catarrhalis.* The carriage rate of viruses was only 7% (17/241), and rhinovirus was the most common species (Table 2).

**Table 2:** Microbial detection in CAP-patients and controls.

Analysis	CAP-patients *n* (%)^b^	Controls *n* (%)^b^	Controls/ CAP-patients tested^b^	*p*
*Streptococcus pneumoniae* – UAD^a^	97 (18.7)	8 (2.0)	396/518	<0.001
*Streptococcus pneumoniae* - BINAX	68 (13.1)	4 (1.0)	396/518	<0.001
*Streptococcus pneumoniae* - NP-culture	21 (5.0)	1 (0.2)	491/420	<0.001
*Haemophilus influenzae* - NP-culture	52 (12.4)	5 (1.0)	491/420	<0.001
*Moraxella catarrhalis* - NP-culture	38 (9.0)	25 (5.1)	491/420	0.019
**Bacterial agents - PCRs**				
*Streptococcus pneumoniae*	85 (17.3)	9 (3.7)	241/490	<0.001
*Haemophilus influenzae*	70 (14.3)	15 (6.2)	241/490	0.001
*Mycoplasma pneumoniae*	28 (5.6)	0	241/502	
*Chlamydia pneumoniae*	1 (0.2)	0	241/490	
*Bordetella parapertussis*	1 (0.2)	0	241/490	
*Bordetella pertussis*	0	0	241/490	
**Viral agents - PCRs**	145 (29.3)	17 (7.1)	241/495^c^	<0.001
Rhino- or Enterovirus	50 (10.1)	10 (4.1)	241/491^c^	0.005
Influenza A	20 (4)	1 (0.4)	241/495^c^	
Influenza B	21 (4.2)	2 (0.8)	241/495^c^	
Human Metapneumovirus	26 (5.3)	0	241/495^c^	
RSV A/B	11 (2.2)	0	241/495^c^	
Parainfluenza virus 1–3	7 (1.4)	1 (0.4)	241/491^c^	
Adenovirus	1 (0.2)	0	241/491^c^	
Coronavirus OC43, NL63, 229E	10 (2.0)	3 (1.2)	241/491^c^	
Parechovirus	0	0	241/491^c^	

a UAD- Urine antigen detection, BINAX – pneumococcal urine antigen, NP- nasopharyngeal, RSV A/B - Respiratory syncytial virus A/B.

b All percentages are calculated from number tested and not the entire cohort.

c Patients (*n* = 490) were tested per protocol, additional patients were tested for some viruses in routine care during the hospital stay.

### Microbial findings in relation to demography

Viruses were a significantly more common finding in male patients, with viruses detected in 35% (97/277) either as a single finding or in combination with bacteria, compared to 22% (47/218) in female patients (*p* = 0.002). In cases where no viruses were detected, males and females were equally represented. Viral detection increased with age, while single bacterial findings were more common in younger patients. Among CAP-patients under 50 years of age, *M. pneumoniae* was the most frequently detected bacterial species (27/59, 46%), while other bacterial species were more evenly distributed in older age groups (Table 3). Patients with viruses were more likely to have coronary heart disease and a higher BMI (Table 4).

**Table 3:** Detected microbes in relation to age groups and clinical symptoms.

	Age group (years)			
	18–49	50–64	65–79	≥ 80
*n* (%)	77 (14.9)	92 (17.8)	187 (36.1)	162 (31.3)
Sex (female)	36 (46.8)	41 (44.6)	91 (48.7)	68 (42.0)
**Microbe**				
*Streptococcus pneumoniae*	22 (28.6)	34 (37.0)	52 (27.8)	39 (24.1)
*Haemophilus influenzae*	7 (9.1)	14 (15.2)	39 (20.9)	24 (14.8)
*Moraxella catarrhalis*	6 (7.8)	8 (8.7)	8 (4.3)	17 (10.5)
*Mycoplasma pneumoniae*	27 (35.1)	1 (1.1)	0 (0)	0 (0)
Enterobacterales*/ P. aeruginosa*^a^	2 (2.6)	1 (1.1)	4 (2.1)	3 (1.9)
*Staphylococcus aureus*	2 (2.6)	1 (1.1)	2 (1.1)	1 (0.6)
*Legionella pneumophila*	0 (0)	2 (2.2)	4 (2.1)	0 (0)
Other bacteria^b^	2 (2.6)	0 (0)	4 (2.1)	1 (0.6)
Other streptococci^c^	2 (2.6)	3 (3.3)	5 (2.7)	1 (0.6)
Rhino−/Enterovirus	1 (1.3)	11 (12.0)	23 (12.3)	15 (9.3)
Human metapneumovirus	3 (3.9)	7 (7.6)	9 (4.8)	7 (4.3)
Influenza B	3 (3.9)	7 (7.6)	6 (3.2)	5 (3.1)
Influenza A H3N2/H1N1	1 (1.3)	2 (2.2)	11 (5.9)	6 (3.7)
RSV A/B^d^	0 (0)	2 (2.2)	4 (2.1)	5 (3.1)
Coronavirus OC43, NL63, 229E	2 (2.6)	2 (2.2)	3 (1.6)	3 (1.9)
Parainfluenza virus 1–3	0 (0)	1 (1.1)	4 (2.1)	2 (1.2)
Adenovirus	1 (1.3)	0 (0)	0 (0)	0 (0)
Parechovirus	0 (0)	0 (0)	0 (0)	0 (0)
**Pathogen detection**				
Bacteria	45 (58.4)	33 (35.9)	57 (30.5)	60 (37.0)
No finding	12 (15.6)	23 (25.0)	64 (34.2)	57 (35.2)
Viral detection	5 (6.5)	11 (12.0)	24 (12.8)	25 (15.4)
Viral-bacterial co-detection	6 (7.8)	20 (21.7)	35 (18.7)	18 (11.1)
**Symptoms/ Clinical findings**				
Fever ≥38.5	70 (90.9)	84 (91.3)	159 (85.0)	125 (77.2)
Chills or rigor	65 (84.4)	64 (69.6)	107 (57.2)	74 (45.7)
Pleuritic chest pain	52 (67.5)	43 (46.7)	69 (36.9)	39 (24.1)
Cough	73 (94.8)	80 (87.0)	161 (86.1)	125 (77.2)
Sputum	55 (71.4)	56 (60.9)	120 (64.2)	80 (49.4)
Dyspnoea	68 (88.3)	71 (77.2)	140 (74.9)	118 (72.8)
Tachypnoea	54 (70.1)	55 (59.8)	106 (56.7)	89 (54.9)
Malaise	67 (87.0)	82 (89.1)	161 (86.1)	143 (88.3)
Auscultatory finding	52 (67.5)	62 (67.4)	139 (74.3)	129 (79.6)

a The group “Enterobacterales/ *P. aeruginosa*” includes *Escherichia coli, Pseudomonas aeruginosa, Enterobacter cloacae, Klebsiella pneumoniae and Proteus mirabilis.*

b The group “Other bacteria” includes *Neisseria meningitidis, Fusobacterium nucleatum* and *Enterococcus faecalis*.

c The group “Other streptococci” includes 10 cases of β-hemolytic streptococci and 1 case of *Streptococcus anginosus.*

d RSV A/B – Respiratory syncytial virus A/B.

**Table 4:** Patient characteristics, symptoms and outcome related to microbes detected.

	CAP-patients grouped by diagnostic finding				
	All	Bacteria	Viruses	Viruses + bacteria	No finding
*n* (%)	518^a^	195 (39.4)	65 (13.1)	79 (16.0)	156 (31.5)
Age, median [IQR]	73 [60–82]	70 [54–83]	76 [65–84]	71 [61–79]	76 [67–83]
Female sex	236 (45.6)	99 (50.8)	23 (35.4)	24 (30.4)	72 (46.2)
Nursing home resident	13 (2.5)	5 (2.6)	1 (1.5)	2 (2.5)	5 (3.2)
Smoker - current	97 (18.7)	39 (20)	11 (16.9)	17 (21.5)	25 (16.1)
Smoker - previous	234 (45.2)	77 (39.5)	35 (53.8)	40 (50.6)	70 (45.2)
BMI, median [IQR]	25 [22–29]	25 [22–28]	28 [24–30]	25 [22–28]	26 [23–29]
**Any Co-morbidity**	359 (69.3)	115 (59.0)	51 (78.5)	62 (78.5)	115 (73.7)
COPD	143 (27.6)	44 (22.6)	17 (26.2)	31 (40.3)	46 (29.7)
Asthma	47 (9.1)	13 (6.7)	8 (12.3)	8 (10.1)	15 (9.7)
Congestive heart disease	95 (18.3)	26 (13.3)	12 (18.5)	14 (17.7)	41 (26.5)
Coronary artery disease	135 (26.1)	41 (21)	27 (41.5)	18 (22.8)	46 (29.7)
Autoimmune disease	32 (6.2)	14 (7.2)	2 (3.1)	3 (3.8)	11 (7.1)
Diabetes	87 (16.8)	25 (12.8)	12 (18.5)	14 (17.7)	32 (20.6)
Liver disease	10 (1.9)	2 (1.0)	0 (0)	4 (5.1)	3 (1.9)
Immunosuppressive therapy ^b^	65 (12.5)	24 (12.4)	9 (13.8)	13 (16.5)	17 (11)
Chronic kidney disease	47 (9.1)	14 (7.2)	7 (10.8)	7 (8.9)	18 (11.6)
Immunodeficiency ^c^	8 (1.5)	2 (1.0)	1 (1.5)	2 (2.5)	2 (1.3)
Cancer - hematologic	19 (3.7)	3 (1.5)	5 (7.7)	8 (10.1)	3 (1.9)
Cancer - solid tumour	106 (20.5)	36 (18.6)	13 (20)	24 (30.4)	32 (20.6)
Pneumococcal vaccine ^d^	56 (10.8)	22 (11.8)	6 (9.5)	5 (6.7)	21 (14.7)
Influenza vaccine ^d^	172 (33.2)	64 (34)	23 (36.5)	27 (36)	52 (35.9)
**Symptoms/ Clinical findings**					
Fever ≥38.5 °C	438 (84.6)	177 (90.8)	50 (76.9)	71 (89.9)	120 (77.4)
Chills or rigor	310 (59.8)	138 (71.1)	30 (46.2)	50 (63.3)	78 (50.3)
Pleuritic chest pain	203 (39.2)	91 (46.7)	17 (26.2)	27 (34.2)	59 (36.8)
Cough	439 (84.7)	173 (88.7)	59 (92.2)	73 (92.4)	113 (72.9)
Sputum	311 (60.0)	125 (64.4)	41 (64)	55 (69.6)	78 (50.3)
Dyspnoea	397 (76.6)	148 (75.9)	50 (76.9)	67 (84.8)	113 (72.9)
Tachypnoea	304 (58.7)	114 (58.5)	38 (58.5)	56 (70.9)	86 (55.5)
Malaise	453 (87.5)	174 (89.2)	55 (84.6)	68 (86.1)	136 (87.7)
CRP mg/L, median [IQR]	132 [58–251]	196 [90–297]	75 [35–146]	146 [77–288]	105 [37–172]
PSI grade IV-V	262 (50.5)	88 (45.4)	39 (60)	47 (59.5)	86 (55.5)
PSI score, mean [SD]	92.6 [35.9]	87.5 [38.4]	97.1 [28.3]	97.7 [30.7]	99.5 [36.2]
CRB-65 3-4	14 (2.7)	3 (1.5)	4 (6.2)	0 (0)	7 (4.5)
Length of stay, median [IQR]	5 [3–8]	5 [3–8]	5 [3–9]	5 [3–8]	6 [3–9]
Intensive care unit admission	14 (2.7)	5 (2.6)	1 (1.5)	0 (0)	3 (1.9)
Case fatality rate 30 days	21 (4.1)	10 (5.1)	3 (4.6)	2 (2.5)	6 (3.8)
Case fatality rate 90 days	43 (8.3)	11 (5.6)	6 (9.2)	4 (5.1)	20 (12.9)

^a^ Twenty-three individuals had no viral diagnostic testing performed and are hence not included at all in any of the infection type groups.

^b^ Prednisolone dose ≥10 mg/day (or equivalent), biological/immunomodulatory or chemotherapy drugs.

^c^ Including organ transplantation, primary immunodeficiency disorders, HIV and AIDS.

^d^ Vaccination status was unknown for 29 individuals regarding pneumococcal vaccination and 24 for influenza.

The case fatality rate (CFR) at 30 (*p* = 0.8) and 90 (*p* = 0.075) days as well as the mean length of stay (*p* = 0.3) did not differ significantly depending on the cause of infection (Table 4). However, men had a significantly higher CFR than women at 30 days (6.4% vs. 1.3%; *p* = 0.003) but not at 90 days (10.6% vs. 5.5%; *p* = 0.051). No significant difference was found in the pneumonia severity index (PSI)-score (*p* > 0.05 in all comparisons) among infections caused by viruses, bacteria, or both (Table 4). CRP levels (mg/L) were significantly higher in patients where bacteria were detected, both with and without concurrent viral detection, compared to the groups with no findings or single viral detection (Supplementary Figure S3).

## Discussion

*Streptococcus pneumoniae* was identified as the leading cause of CAP, followed by *H. influenzae*. Rhinovirus was the most prevalent virus and was frequently found in conjunction with bacteria. Establishing an etiology in CAP-patients is challenging. We included samples from routine care as well as per-protocol testing, using a comprehensive range of diagnostic assays including culture, real-time PCR, and urinary antigens. The overall diagnostic yield, encompassing both viral and bacterial pathogens, was 68%, which falls within the range reported in previous studies (38–87%) (6, 10–14).

Regarding *S. pneumoniae* our results align with other European studies, where 29–42% of hospitalized CAP patients were found to be infected with pneumococci (3, 6, 12–15). These numbers, however, are higher than the detection rates of 8–12% reported in recent American studies (10, 16). It has been suggested that widespread pneumococcal vaccination in adults, along with the decline in cigarette smoking in the US, could be contributing factors to the lower detection rates observed (4). Furthermore, it is worth noting that none of the American studies included upper respiratory samples. Although smoking prevalence is lower in Sweden compared to the US (17, 18), *S. pneumoniae* was still detected in 127 (25% of our cohort), even after excluding positive NP-samples.

A previous study by Johansson et al. (13) reported a higher prevalence of *S. pneumoniae* (38%) in adult CAP patients before the introduction of PCV in the Swedish child immunization program, compared to 28% in our study. However, their diagnostic methods involved a higher usage of sputum samples, and they did not have access to UAD, which makes direct comparison challenging.

*Haemophilus influenzae* was identified in 84 cases (16%) and emerged as the second most detected pathogen. Results from other studies show considerable variation, ranging from almost absent with detection rates ≤5% (11, 12) to recent studies where *H. influenzae* was identified as the predominant bacterial etiology with detection rates of 16–40% (5, 6). However, it is important to note that earlier studies did not include DNA detection. Both German and Danish studies have observed increased rates of *H. influenzae* in CAP over the past decade (19, 20). Additionally, an increased carriage rate of non-typeable *H. influenzae* has been noticed in children following the introduction of PCV13 in the child immunization program (21), and certain strains have exhibited increased invasive potential (22), which may explain this phenomenon. These findings highlight the need for further surveillance as an increased incidence of *H. influenzae* in CAP may necessitate changes in treatment guidelines (23). The prevalence of *M. catarrhalis* was similar in both controls and CAP-patients, with rates of 5 and 9%, respectively, as reported by Lieberman et al. (24). These findings suggest that the presence of *M. catarrhalis* in NP samples should be interpreted with caution.

In the tested patients, viruses were found in 29% of cases. Other studies have reported viral detection rates ranging from 15 to 57%, with rhinovirus or influenza virus being the most frequently identified (6, 10, 11, 14). In our study, rhinovirus and hMPV were detected in 10% (*n* = 50) and 5% (*n* = 26) of patients, respectively. It is important to note that all but two of these cases were missed in routine diagnostic testing highlighting the underestimation of viruses in CAP in routine care. Testing for influenza virus and initiating antiviral therapy has been shown to reduce antibiotic usage and improve patient outcomes (25). In the control group, a virus was detected in 7% of cases, with rhinovirus being the most common. This finding could be attributed to asymptomatic carriage or persistence of viral RNA from a previous infection. Previous studies have found rhinovirus in asymptomatic controls, albeit at lower rates (11).

In the present study, 55% of all viral detections were found in combination with bacteria. When comparing single pathogen findings to viral-bacterial co-detection, we found no significant difference in mortality rates. Furthermore, there were no significant differences observed in the length of hospital stay or PSI-score. Previous studies have reported higher proportions of viral-bacterial etiologies in ICU patients (3, 26), but no significant differences in mortality rates (6, 27), and varying results in PSI-score and length of stay (10, 27, 28).

It is known that male sex is a risk factor for CAP (29). However, interestingly, in our investigation males were only predominant in the groups with viral pathogen detection. When only bacteria were identified, both sexes were equally represented. As observed previously (26), single bacterial findings were more common in younger patients, whereas viruses or the absence of any detected pathogen, was more common among older patients. This finding may be explained by a lower threshold for hospitalization of older patients. In our study, symptoms were not a reliable predictor in distinguishing between etiology. All symptoms, except malaise, decreased with age, highlighting the need for increased awareness of CAP in older adults and the possibility of reducing antibiotic treatment with increased sampling in this group. CRP levels were generally high in hospitalized patients with radiographically confirmed CAP, with a median level of 132 mg/L. However, significantly higher levels were observed in patients with bacterial detection compared to those with no finding or single viral detection. Additionally, CRP levels were significantly higher in patients with co-detection of bacteria and viruses compared to those with single viral findings. This indicates that CRP levels below 200 mg/L do not discriminate bacterial pneumonia or superinfection from sole viral infection, and further studies on reliable predictors to support decisions to refrain from antibiotics are needed. Furthermore, we only registered the first recorded CRP value, which means that we do not know whether patients had a higher value later during their hospital stay. However, this addresses the challenge of deciding on empirical treatment in the emergency room.

The absence of a gold standard poses a difficulty in determining the accuracy of diagnostic tests in CAP. However, sampling from the lower respiratory tract has been preferred. In our study, only 12% of the patients had lower respiratory tract samples taken, reflecting the challenge of obtaining high-quality sputum samples prior to antibiotic administration, as reported in other studies (10, 11). We included nasopharyngeal culture, as recommended in Swedish guidelines (23), when sputum was not obtainable. Additionally, PCRs for *S. pneumoniae* and *H. influenzae* were included. Several recent studies have shown the diagnostic value of using PCR for diagnosing CAP using NP swabs (5, 30).

To address concerns regarding colonization of the upper respiratory tract, we included a control group. *Streptococcus pneumoniae* was detected by nasopharyngeal culture or PCR in 4% of the controls, compared to 17% of the study patients. *Haemophilus influenzae* was relatively more common in the controls, with a prevalence of 6% compared to 16% in patients with CAP. Since PCR was not quantitative, it was challenging to establish a cut-off for excluding individuals with only carriage. However, PCR still detected most cases.

Our study has several strengths, including a well-defined cohort, a high frequency of per-protocol samples, and a continuously enrolled control group covering the entire study period. However, the study also has limitations. Obtaining consent from severely ill patients was more difficult, which may have resulted in a somewhat healthier study population compared to the overall cohort of hospitalized CAP patients. The nasopharyngeal samples for PCR were collected after antibiotic administration, but since PCR can detect dead bacteria, it should not be affected by antibiotic use. Since molecular testing, except for atypical bacteria, only included *S. pneumoniae* and *H. influenzae*, these bacteria were more likely to be detected. Additionally, most positive cases for *S. pneumoniae* were identified through urinary antigens, a method only available for pneumococci and *L. pneumophila*. For the latter, only 36% were tested, potentially resulting in missed cases. Despite our efforts, we were unable to identify a pathogen in 33% of cases, which could lead to an underestimation of either viral or bacterial agents.

In conclusion, *S. pneumoniae* was the most common finding, followed by *H. influenzae* and viruses, symptoms and outcomes were similar regardless of etiology. Routine diagnostics underestimated all findings especially viruses which were particularly common in men. Further research is needed to understand the importance of viruses in CAP. Development and implementation of rapid diagnostic tests for both bacteria and viruses could help in distinguishing between viral and bacterial CAP to avoid over-use of antibiotics. The high numbers of *H. influenzae* indicates possible shifts in etiology which should be monitored. Finally, our results suggest that improving the coverage of pneumococcal and influenza vaccines for targeted risk groups, as well as the development of new antiviral treatments for respiratory viruses, could potentially decrease the morbidity of CAP.

## Data availability statement

The original contributions presented in the study are included in the article/Supplementary material, further inquiries can be directed to the corresponding author.

## Ethics statement

The studies involving humans were approved by Ethical approval was granted by the Lund University regional ethics committee (approval nos: 2016/220 and 2016/340), and written informed consent was obtained from all patients. The studies were conducted in accordance with the local legislation and institutional requirements. Written informed consent for participation was not required from the participants or the participants' legal guardians/next of kin in accordance with the national legislation and institutional requirements.

## Author contributions

KH: Conceptualization, Data curation, Formal Analysis, Investigation, Methodology, Project administration, Writing – original draft, Writing – review & editing. LYY: Conceptualization, Data curation, Formal Analysis, Investigation, Methodology, Project administration, Visualization, Writing – original draft, Writing – review & editing. LW: Methodology, Project administration, Resources, Supervision, Writing – review & editing. ER: Data curation, Methodology, Project administration, Writing – review & editing. TG: Data curation, Methodology, Writing – review & editing. AN: Methodology, Supervision, Writing – review & editing. JA: Conceptualization, Funding acquisition, Supervision, Writing – review & editing. KR: Conceptualization, Funding acquisition, Methodology, Resources, Supervision, Writing – review & editing.
