# Supplementary material for: Exploring the microbial landscape: uncovering the pathogens associated with community-acquired pneumonia in hospitalized patients
Source: Front Public Health. 2023 Dec 13;11:1258981. doi: 10.3389/fpubh.2023.1258981 (PMC10752608; doi:10.3389/fpubh.2023.1258981)
Supplement: Supplementary file 1 [file Data_Sheet_1.pdf]

Supplementary material

Exploring the microbial landscape: Uncovering the pathogens associated with community-acquired pneumonia in hospitalized patients

Karin Hansen, MD[1,2†], Linda Yamba Yamba, MD[1†], Lisa Wasserstrom, PhD[1,3], Elisabeth Rünow, MD[1,2], Tommy Göransson, Bsc[3], Anna Nilsson, MD[2], Jonas Ahl, MD[1,2] and Kristian Riesbeck, MD[1,3, *]

[1] Clinical Microbiology and [2] Infectious Diseases, Department of Translational Medicine, Faculty of Medicine, Lund University, Malmö, Sweden

[3] Clinical Microbiology, Infection Control and Prevention, Laboratory Medicine, Lund, Sweden

† These authors share first authorship

* Corresponding author: Kristian Riesbeck, Clinical Microbiology, Dept. of Translational Medicine, Faculty of Medicine, Lund University, Jan Waldenströms gata 59, SE21428 Malmö, Sweden. Phone: +46-40-338494, mobile: +46-(0)730-377433. *E-mail address*: kristian.riesbeck@med.lu.se

Methods

Legionella Urine antigen test

Legionella urine antigen test were analysed at the local laboratory stated above. The laboratory test was changed during the study period, first Alere BinaxNow were used and later ImmuView. Both are rapid in vitro immunochromatographic assays for detection of Legionella pneumophila serogroup 1 antigen.

BinaxNOW S. pneumoniae® / Urine antigen detection (UAD)

Urine testing BinaxNOW *S. pneumoniae*® detects pneumococcal C-polysaccharide (C-PS) antigens in the urine using an immunochromatographic membrane test kit. The UAD test is a limit assay that uses Luminex technology, with positivity cut-off limits based on antigen concentrations read off a standard curve. UAD1 and 2 combined detects 24 *S. pneumoniae* serotype-specific polysaccharides (serotypes 1, 3, 5, 6A, 6B, 7F, 9V, 14, 18C, 19A, 19F 23F, 2, 8, 9N, 10A, 11A, 12F, 15B/C, 17F, 20, 22F and 33F).[1,2]

Viral and bacterial detection by real-time PCR

The nasopharyngeal samples were stored at -80 ºC at the Department of Clinical Microbiology, Lund prior to real-time PCR analysis. DNA/RNA was extracted from 200 µl sample without pretreatment, using the MagNA Pure 96 DNA and Viral NA Small volume kit (Roche Diagnostics, Basel, Switzerland) with addition of 20 µl poly(A) (Roche Diagnostics, Basel, Switzerland) per sample. The elution volume was 100 µl. RT Realtime-PCR amplifications for viral agents were performed on an ABI 7500 real-time PCR system (Applied Biosystems, Waltham, USA) in five multiplex reactions, adopted from Østby et al., and Ek et al.,[3, 4] containing; 1) influenza A including subtyping of H1N1/H3N2 and influenza B, 2) respiratory syncytical virus (RSV) A/B and human metapneumovirus (hMPV), 3) parainfluenza virus (PIV) 1 to 3 and adenovirus, 4) coronavirus (OC43, NL63, 229E) and rhinovirus, 5) enterovirus and parechovirus. For all five reactions the Path-ID Multiplex One-Step RT-PCR Kit (Ambion, Life Technologies, Carlsbad, USA) was used and the following PCR-program: a reverse transcription step at 48 ºC for 10 minutes and a RT-inactivation and initial denaturation step at 95 ºC for 10 minutes followed by 45 cycles of PCR-amplification at 95 ºC for 15 seconds and 55 ºC for 45 seconds. Samples generating a detectable signal with a S-shaped amplification plot were classified as positive for the analysed viral target. Real-time PCR amplification for bacterial detection were performed using SensiFAST Probe No-ROX Kit (Bioline, Meridian Bioscience, Cincinnati, USA) and a Bio-Rad CFX96 (Bio-Rad, Hercules, USA) in the following singleplex and multiplex reactions: 1) *Haemophilus influenzae*, 2) *S. pneumoniae*, 3) *Bordetella parapertussis* and *B. pertussis*, 4) *Mycoplasma pneumoniae* and *Chlamydia pneumoniae*. For reaction 1 and 2 the following PCR-program was used: an initial denaturation step at 95 ºC for 3 minutes followed by 45 cycles of PCR-amplification at 95 ºC for 10 seconds and 60 ºC for 50 seconds. For reaction 3 and 4 the following PCR-program was used: an initial denaturation step at 95 ºC for 5 minutes followed by 45 cycles of PCR-amplification at 95 ºC

for 10 seconds and 60 ºC for 30 seconds. The PCR for *S. pneumoniae* was an in house method developed at the Clinical Microbiology Department (Lund) using forward primer LytA-1-F 5'-GCTGGGTCAAGTACAAGGACACT-3, reverse primer LytA-1-R 5'-GTCCGCTGACTGGATAAAGGCA-3' and probe Lyt-P 5'- FAM-ACTTAGACGCTAAAGAAGGCGCCATGGTATC-BHQ-1-3'.The PCR for a) *H. influenzae* was adopted from Smith-Vaughan *et al.*[5], b) *M. pneumoniae* was adopted from Hardegger *et al.*[6], c) *C. pneumoniae* from Welti *et al.*[7], and d) *B. pertussis* and *B. parapertussis* was adopted from Roorda *et al.*[8],. A sample was considered positive if the Cq-value was below cut-off for the specific PCR and had amplification curves indicating efficient PCR. Bacterial samples were rerun to confirm a positive test if PCR-curves were inconsistent i.e., low amplification, not S-shaped, as reviewed by the interpreter although Cq values were below cut-off. If results could be repeated from another extraction the isolate was considered positive and otherwise negative.

Bacterial cultures

Blood, respiratory tract specimens and pleural fluid were cultured according to standard methods at Clinical Microbiology Lab at Laboratory Medicine Skåne. Upper and lower respiratory tract samples were cultured on agar plates in aerobic, anaerobic and CO_2 conditions at 33-37°C. Blood cultures were performed using the automatic BACTEC system (BD diagnostic systems, Sparks, MD). Colony morphology together with standard biochemical tests and MALDI Biotyper analysis (Bruker Daltonics, Bremen, Germany) was used for species identification. [9,10,11]

Supplementary References

1. Pride MW, Huijts SM, Wu K, et al. Validation of an immunodiagnostic assay for detection of 13 Streptococcus pneumoniae serotype-specific polysaccharides in human urine. *Clin Vaccine Immunol* 2012; **19**(8): 1131-41.
2. Kalina WV, Souza V, Wu K, et al. Qualification and Clinical Validation of an Immunodiagnostic Assay for Detecting 11 Additional Streptococcus pneumoniae Serotype-specific Polysaccharides in Human Urine. *Clin Infect Dis* 2020; **71**(9): e430-e8.
3. Østby AC, Gubbels S, Baake G, Nielsen LP, Riedel C, Arpi M. Respiratory virology and microbiology in intensive care units: a prospective cohort study. *Apmis* 2013; **121**(11): 1097-108.
4. Ek P, Böttiger B, Dahlman D, Hansen KB, Nyman M, Nilsson AC. A combination of naso- and oropharyngeal swabs improves the diagnostic yield of respiratory viruses in adult emergency department patients. *Infect Dis (Lond)* 2019; **51**(4): 241-8.
5. Smith-Vaughan H, Byun R, Nadkarni M, et al. Measuring nasal bacterial load and its association with otitis media. *BMC Ear Nose Throat Disord* 2006; **6**: 10.
6. Hardegger D, Nadal D, Bossart W, Altwegg M, Dutly F. Rapid detection of Mycoplasma pneumoniae in clinical samples by real-time PCR. *J Microbiol Methods* 2000; **41**(1): 45-51.
7. Welti M, Jaton K, Altwegg M, Sahli R, Wenger A, Bille J. Development of a multiplex real-time quantitative PCR assay to detect Chlamydia pneumoniae, Legionella pneumophila and Mycoplasma pneumoniae in respiratory tract secretions. *Diagn Microbiol Infect Dis* 2003; **45**(2): 85-95.
8. Roorda L, Buitenwerf J, Ossewaarde JM, van der Zee A. A real-time PCR assay with improved specificity for detection and discrimination of all clinically relevant Bordetella species by the presence and distribution of three Insertion Sequence elements. *BMC Research Notes* 2011; **4**(1): 11.
9. Jorgensen JH, Pfaller MA, Carroll KC. Manual of clinical microbiology. 11th edition. Washington, DC: ASM Press; 2015.
10. Claesson B, Hallander H, Nyberg A, et al. Referensmetodik för laboratoriediagnostik vid kliniskt mikrobiologiska laboratorier. Referensmetodik I 2 Nedre luftvägsinfektioner. 2 ed: Föreningen för Klinisk Mikrobiologi vid Svenska Läkaresällskapet med stöd av Folkhälsomyndigheten; 2005.
11. Bergquist S, Hallander H, Holm S, et al. Referensmetodik för laboratoriediagnostik vid kliniskt mikrobiologiska laboratorier. Referensmetodik: Övre luftvägsinfektioner (ÖLI). Smittskyddsinstitutet; 1995.

Supplementary Figure 1. Study profile. Flow chart resulting in 518 of study patients included in the final analyses.

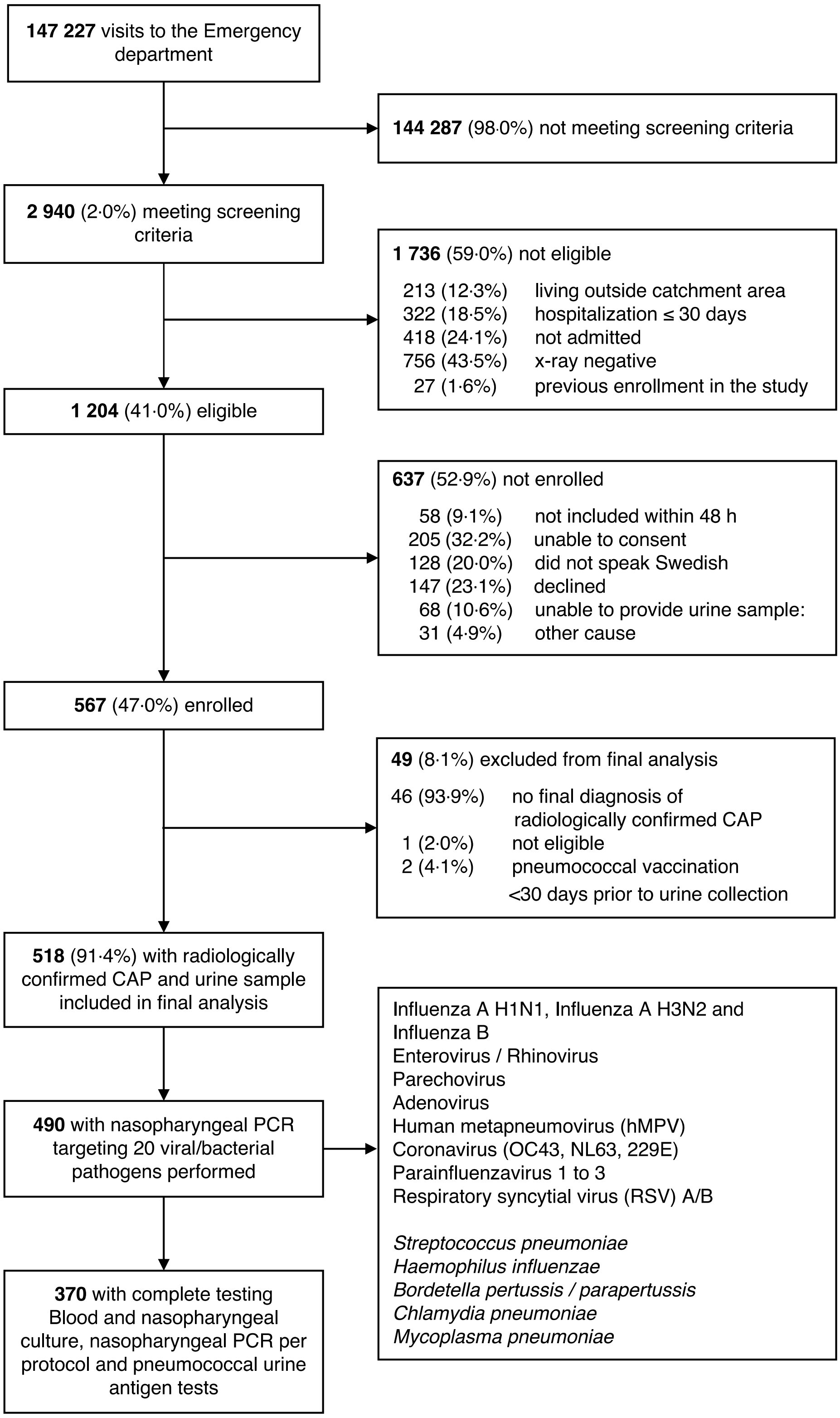

Supplementary Figure 2. Seasonal variation of major bacterial and viral pathogens is depicted. In **(A)** the number of cases per month for *S. pneumoniae*, *H. influenzae* and *M. pneumoniae* is shown from September 2016 to September 2018. In **(B)**, Rhino-/ enterovirus, influenza A and B, respiratory syncytial virus (RSV A/B), and human metapneumovirus (hMPV) are indicated for the same period.

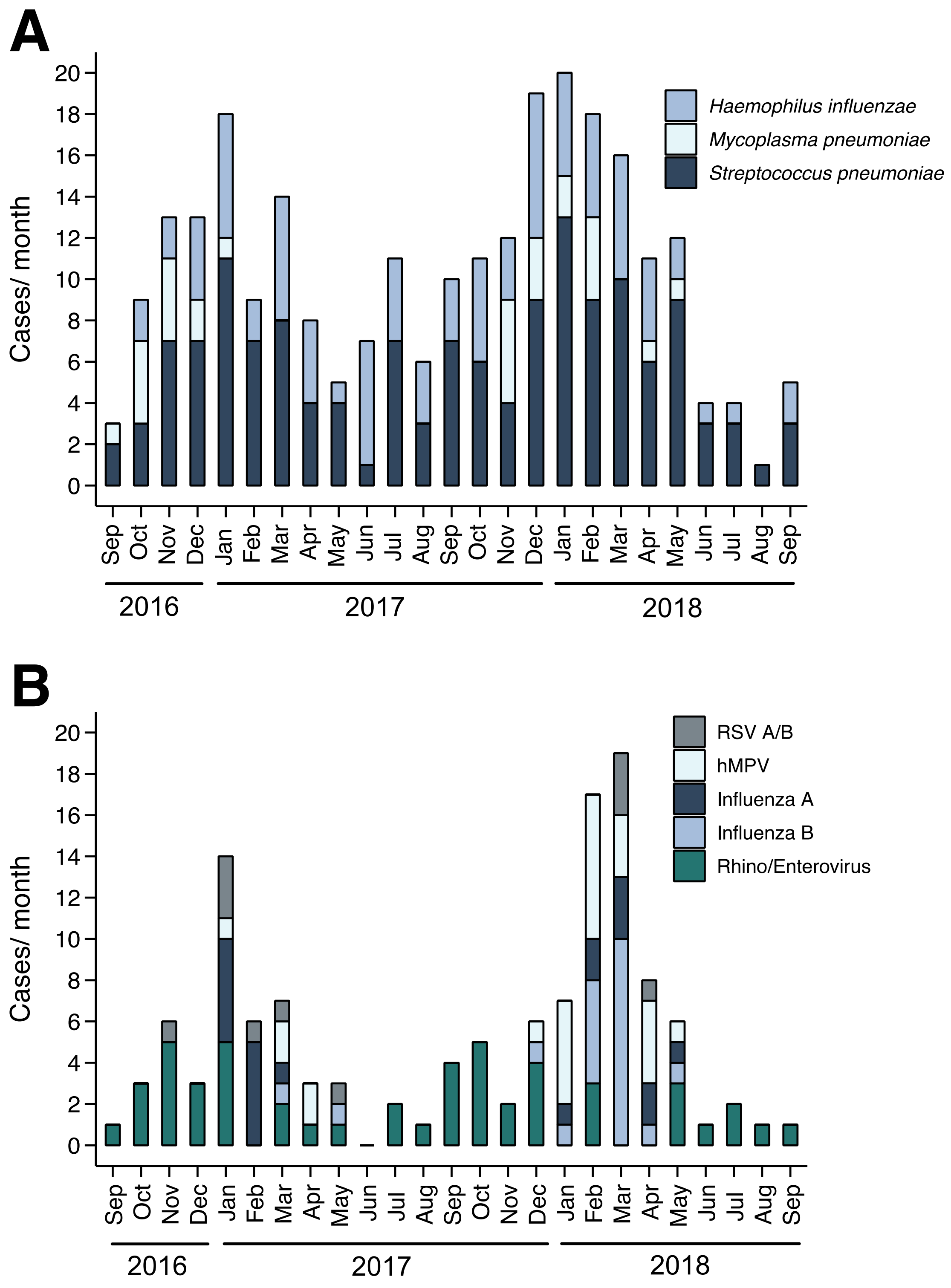

Supplementary Figure 3. CRP levels at admission depending on pathogen detection. Significantly higher CRP levels (mg/L) were observed in the groups with detection of bacteria (median = 196, *n*=194) or bacteria + viruses (median=146, *n*=79) compared to single viral infection (median=75, *n*=64) and no finding (median=105, *n*=154). Four cases with unavailable CRP were excluded from the analysis.

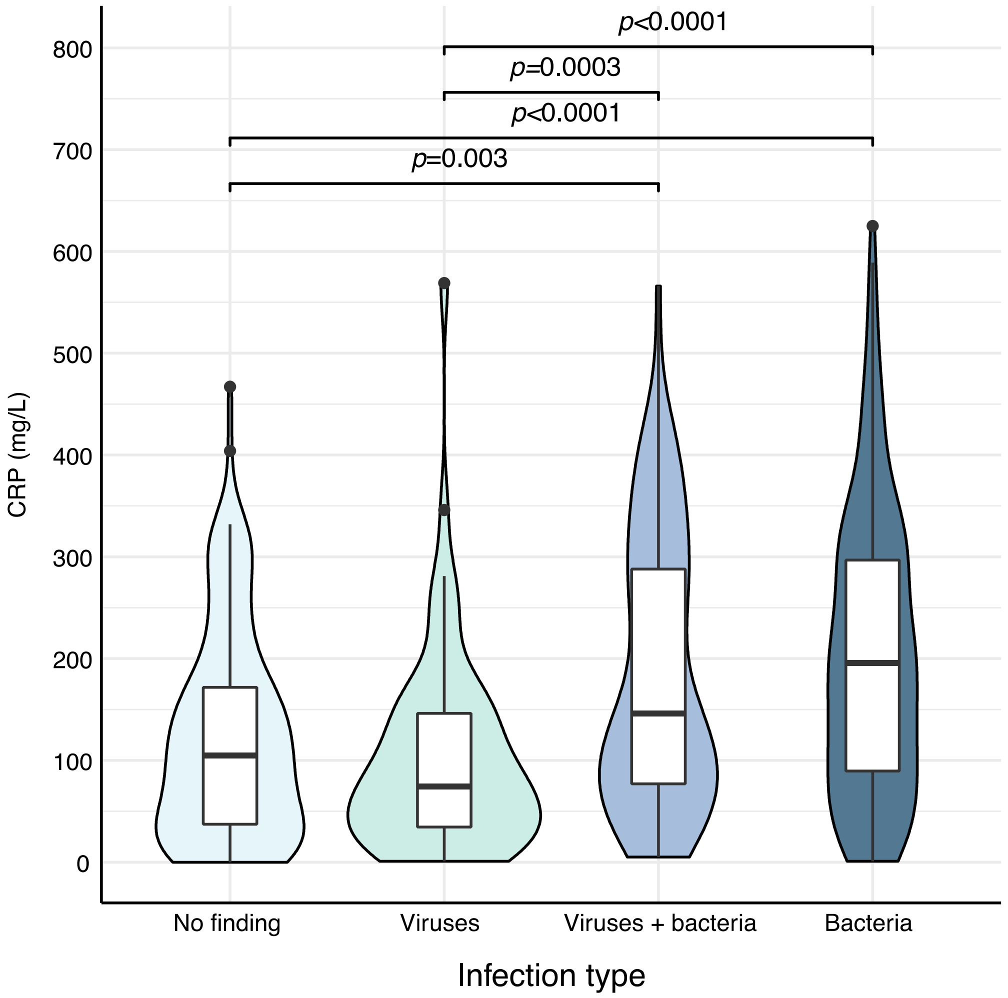

Supplementary Table 1. Detection of pathogens, unique cases within the cohort combining all diagnostic methods.

	**Controls (*n*=241)**	**Subjects (*n*=518)**
No finding	190 (78.8)	167 (32.2)
**Bacteria**		
Streptococcus pneumoniae	15 (6.2)	147 (28.4) [a]
Haemophilus influenzae	15 (6.2)	84 (16.2)
Moraxella catarrhalis	16 (6.6)	39 (7.5)
Staphylococcus aureus	-	6 (1.2)
Enterobacterales / *P. aeruginosa*	-	10 (1.9)
Other streptococci	1 (0.4)	11 (2.1)
Other bacteria	-	5 (1.0)
**Atypical bacteria**		
Mycoplasma pneumoniae	0	28 (5.4)
Chlamydophila pneumoniae	0	1 (0.2)
Bordetella parapertussis	0	1 (0.2)
Bordetella pertussis	0	0
Legionella pneumophila	-	6 (1.2%)
**Viral**		
Virus detected	17 (7.1)	144 (28)
Influenza A H3N2/H1N1	1 (0.4)	20 (3.9)
Influenza B	2 (0.8)	21 (4.0)
RSV A/B	0	11 (2.1)
hMPV	0	26 (5.0)
Rhino-/enterovirus [b]	10 (4.1)	50 (9.7)
Corona NL63	0	4 (0.8)
Corona 229E	2 (0.8)	2 (0.4)
Corona OC43	1 (0.4)	4 (0.8)
Adenovirus	0	1 (0.2)
PIV1	1 (0.4)	1 (0.2)
PIV2	0	0
PIV3	0	6 (1.2)
Parechovirus	0	0

[a] = Only 2 additional cases were added to the subjects by adding the results from blood cultures and lower respiratory tract diagnostic tests.
[b] = All cases were rhinovirus 10/10 among controls. Among subjects 45/50 cases were rhinovirus, 1 enterovirus and 4 could not be determined if rhino or enterovirus.
(-) = not tested for among controls

Supplementary Table 2. Distribution of bacteria and viruses detected.

Bacteria *n* (%)	Viruses: No detected virus	Entero/ Rhinovirus [d]	Influenza A/ B	hMPV	RSV	Corona OC43/ NL63/22E	PIV 1-3 [e]	Other viruses[f]	**Total**
No detected bacteria	167 (32.2)	15 (2.9)	23 (4.4)	15 (2.9)	5 (1.0)	3 (0.6)	3 (0.6)	1	235 (45.4)
**Single bacteria**									
S. pneumoniae	76 (14.7)	18 (3.5)	7 (1.4)	5 (1.0)	5 (1.0)	3 (0.6)	1 (0.2)	2 (0.4)	117 (22.6)
H. influenzae	43 (8.3)	9 (1.7)	3 (0.6)	1 (0.2)	0	0	0	0	56 (10.8)
M. pneumoniae	22 (4.2)	0	0	1 (0.2)	0	0	0	0	20 (3.9)
M. catarrhalis	16 (3.1)	0	1 (0.2)	1 (0.2)	0	1 (0.2)	0	0	19 (3.7)
L. pneumophila	5 (1.0)	0	1 (0.2)	0	0	0	0	0	6 (1.2)
S. aureus	4 (0.8)	0	0	0	0	0	1 (0.2)	0	5 (1.0)
β-hemolytic streptococci	3 (0.6)	1 (0.2)	1 (0.2)	1 (0.2)	0	0	0	0	6 (1.2)
Other [a]	7 (1.4)	1 (0.2)	0	0	0	0	0	0	8 (1.5)
**Multiple bacteria**									
S. pneumoniae + *H. influenzae*	9 (1.7)	2 (0.4)	1 (0.2)	1 (0.2)	0	0	1 (0.2)	0	14 (2.7)
S. pneumoniae + *M catarrhalis*	6 (1.2)	1 (0.2)	1 (0.2)	1 (0.2)	0	1 (0.2)	0	0	10 (1.9)
M. pneumoniae + *H. influenzae, S. pneumoniae* or *M. catarrhalis* [b]	5 (1.0)	0	0	0	0	0	0	0	5 (1.0)
S. pneumoniae + streptococci [c]	3 (0.6)	0	0	0	0	0	0	0	3 (0.6)
H. influenzae + *M catarrhalis*	2 (0.4)	1 (0.2)	0	0	0	0	1 (0.2)	0	4 (0.8)
H. influenzae + Other [a]	3 (0.6)	0	0	0	0	1 (0.2)	0	0	4 (0.8)
≥ 3 different bacteria	3 (0.6)	0	1 (0.2)	0	0	0	0	0	4 (0.8)
M. catarrhalis + Other [a]	1 (0.2)	1 (0.2)	0	0	0	0	0	0	2 (0.4)
M catarrhalis+ β-h*emolytic streptococci*	0	0	1 (0.2)	0	0	0	0	0	1 (0.2)
**Total**	373	49	41	26	10	9	7	3	518

[a] Including Gram-negative bacteria (*Escherichia coli, Klebsiella pneumoniae, Pseudomonas aeruginosa)* 1 case of *Bordetella parapertussis* and *Chlamydia pneumoniae* respectively.
[b] 3 cases of *Haemophilus influenzae*, 1 *Streptococcus pneumoniae* and 1 *Moraxella catarrhalis.*
[c] 2 cases of β-hemolytic *streptococci* and 1 case *Streptococcus anginosus.*
[d] 44 cases rhinovirus, 1 case enterovirus, 4 undetermined.
[e] Parainfluenza virus.
[f] 1 case of adenovirus, 2 cases of dual viruses: RSV + corona NL63 and rhino-/ enterovirus + influenza A.

Supplementary Table 3. Overview of viral diagnostic testing in the cohort. The per protocol PCR tests found additional cases of several viruses like influenza, RSV, hMPV and rhino/enterovirus not detected during the hospital stay. Two individuals carried two viruses. In the cohort, 4.4% (*n*=23) had no viral diagnostic test performed either during their hospital stay or had nasopharyngeal samples available for the additional PCR.

**Viral diagnostic testing**	**Study** Extended Multiplex[b] *n* (%)	**Routine diagnostics n (% of tested)** Rapid PCR *n* (%)	Multiplex *n* (%)	Extended Multiplex[c] *n* (%)	Unique findings (*n*)	Tested individuals *n* (%)
**Tested**	490	133	21	19		495
**Virus detected n (%)**	135 (27.6)	29 (21.8)	14 (67.7)	4 (21.1)	146	144 (29)
**Influenza A**	13 (2.7)	10 (7.5)	11 (52.4)	1 (5.3)	20	(4.0)
**Influenza B**	20 (4.1)	12 (9.0)	1 (4.8)	1 (5.3)	21	(4.2)
**RSV A/B**	11 (2.2)	7 (5.2)	0	0	11	(2.2)
**hMPV**	26 (5.3)	-	2 (9.5)	0	26	(5.3)
**Rhino-/ enterovirus[a]**	50 (10.2)	-	-	1 (5.3)	50	(10.1)
**CoronaNL63**	4 (0.8)	-	-	0	4	(0.8)
**Corona229E**	2 (0.4)	-	-	1 (5.3)	2	(0.4)
**CoronaOC43**	4 (0.8)	-	-	0	4	(0.8)
**Adenovirus**	1 (0.2)	-	-	0	1	(0.2)
**PIV1**	1 (0.2)	-	-	0	1	(0.2)
**PIV2**	0	-	-	0	0	0
**PIV3**	6 (1.2)	-	-	0	6	(1.2)
**Parechovirus**	0	-	-	0	0	0

[a] The method could not always distinguish between rhinovirus and enterovirus. 45 rhinovirus, 1 enterovirus, 4 undistinguishable.

[b] Per protocol samples.

[c] Routine care testing.

Supplementary Table 4. Overview of bacterial diagnostic testing in the cohort. The use of UAD and PCR, not available in routine diagnostics, increased detection of both *S. pneumoniae* and *H. influenzae*. PCR for *S. pneumoniae* and *H. influenzae* added 19 and 23 additional cases respectively. Not all tests were performed for all study subjects.

**Bacterial diagnostic testing (%)**	**PCR per protocol**	**PCR routine care**	**Urine antigen**			**Culture**					**Other tests**[c]	**All tests**
	NP	NP / OP[a]	UAD	BINAX-pnc	Legionella	NP	Blood	Sputum	Other LRT[b]	Pleural		Unique cases
n tested	490	136	518	518	186	420	470	28	14	16		
Negative		108 (79.4)	421 (81.3)	450 (86.9)	181 (97.3)	312 (74.3)	435 (92.6)	19 (67.9)	5 (35.7)	11 (68.8)		
S. pneumoniae	85 (17.3)	-	97 (18.7)	68 (13.1)	-	21 (5)	22 (4.7)	1 (3.6)	1 (7.1)	0		147 (28.4)
H. influenzae	70 (14.3)	-	-	-	-	52 (12.4)	2 (0.4)	2 (7.1)	1 (7.1)	0	1	84 (16.2)
M. catarrhalis	-	-	-	-	-	38 (9)	0	2 (7.1)	0	0		39 (7.5)
S. aureus	-	-	-	-	-	-	3 (0.6)	2 (7.1)	3 (21.4)	1 (6.3)		6 (1.2)
Enterobacterales / *P. aeruginosa* [d]	-	-	-	-	-	-	3 (0.6)	5 (17.9)	4 (28.6)	1 (6.3)		10 (1.9)
Other streptococci [d]	-	-	-	-	-	6 (0.6)	3 (0.6)	0	1 (7.1)	2 (12.5)	1	11 (2.1)
Other bacteria [d]	-	-	-	-	-	-	1 (0.2)	0	2 (14.3)	1 (6.3)	1	5 (1)
**Atypical bacteria**												
M. pneumoniae	20 (4.1)	27 (19.9)	-	-	-	-	-	-	-	-	2	28 (5.4)
C. pneumoniae	0	1 (0.7)	-	-	-	-	-	-	-	-		1 (0.2)
B. parapertussis	1 (0.2)	-	-	-	-	-	-	-	-	-		1 (0.2)
B. pertussis	0	-	-	-	-	-	-	-	-	-		0
L. pneumophila	-	-	-	-	5 (2.7)	-	-	-	-	-	4	6 (1.2)

[a] Patients were sampled with oropharyngeal (OP) and/or nasopharyngeal (NP) swabs. Patients positive in both OP and NP have been calculated only once.
[b] LRT= Lower respiratory tract. Bronchial alveolar lavage/sterile brush or tracheal aspirate.
[c] PCR or 16S rDNA from samples from pleural fluid or LRT.
[d] The group Other bacteria includes *Neisseria meningitidis*, *Fusobacterium nucleatum* and *Enterococcus faecalis*.
The group "Enterobacterales */ P aeruginosa*" includes *Escherichia coli, Pseudomonas aeruginosa, Enterobacter cloacae, Klebsiella pneumoniae* and *Proteus mirabilis.*
The group "Other streptococci" includes 10 cases of β-hemolytic streptococci 1 case of *Streptococcus anginosus.*
